# Ionizing Radiation Protein Biomarkers in Normal Tissue and Their Correlation to Radiosensitivity: A Systematic Review

**DOI:** 10.3390/jpm11020140

**Published:** 2021-02-19

**Authors:** Prabal Subedi, Maria Gomolka, Simone Moertl, Anne Dietz

**Affiliations:** Bundesamt für Strahlenschutz/Federal Office for Radiation Protection, Ingolstädter Landstraße 1, 85764 Oberschleissheim, Germany; mgomolka@bfs.de (M.G.); smoertl@bfs.de (S.M.); adietz@bfs.de (A.D.)

**Keywords:** ionizing radiation, normal tissue, biomarker, radiotherapy, radiosensitivity, proteomics

## Abstract

**Background and objectives**: Exposure to ionizing radiation (IR) has increased immensely over the past years, owing to diagnostic and therapeutic reasons. However, certain radiosensitive individuals show toxic enhanced reaction to IR, and it is necessary to specifically protect them from unwanted exposure. Although predicting radiosensitivity is the way forward in the field of personalised medicine, there is limited information on the potential biomarkers. The aim of this systematic review is to identify evidence from a range of literature in order to present the status quo of our knowledge of IR-induced changes in protein expression in normal tissues, which can be correlated to radiosensitivity. **Methods**: Studies were searched in NCBI Pubmed and in ISI Web of Science databases and field experts were consulted for relevant studies. Primary peer-reviewed studies in English language within the time-frame of 2011 to 2020 were considered. Human non-tumour tissues and human-derived non-tumour model systems that have been exposed to IR were considered if they reported changes in protein levels, which could be correlated to radiosensitivity. At least two reviewers screened the titles, keywords, and abstracts of the studies against the eligibility criteria at the first phase and full texts of potential studies at the second phase. Similarly, at least two reviewers manually extracted the data and accessed the risk of bias (National Toxicology Program/Office for Health Assessment and Translation—NTP/OHAT) for the included studies. Finally, the data were synthesised narratively in accordance to synthesis without meta analyses (SWiM) method. **Results**: In total, 28 studies were included in this review. Most of the records (16) demonstrated increased residual DNA damage in radiosensitive individuals compared to normo-sensitive individuals based on γH2AX and TP53BP1. Overall, 15 studies included proteins other than DNA repair foci, of which five proteins were selected, Vascular endothelial growth factor (VEGF), Caspase 3, p16^INK4A^ (Cyclin-dependent kinase inhibitor 2A, CDKN2A), Interleukin-6, and Interleukin-1β, that were connected to radiosensitivity in normal tissue and were reported at least in two independent studies. **Conclusions and implication of key findings**: A majority of studies used repair foci as a tool to predict radiosensitivity. However, its correlation to outcome parameters such as repair deficient cell lines and patients, as well as an association to moderate and severe clinical radiation reactions, still remain contradictory. When IR-induced proteins reported in at least two studies were considered, a protein network was discovered, which provides a direction for further studies to elucidate the mechanisms of radiosensitivity. Although the identification of only a few of the commonly reported proteins might raise a concern, this could be because (i) our eligibility criteria were strict and (ii) radiosensitivity is influenced by multiple factors. **Registration**: PROSPERO (CRD42020220064).

## 1. Introduction

### 1.1. Background and Rationale

Ionizing radiation is increasingly applied in medical therapy and diagnosis procedures. IARC Global Cancer Observatory reports more than 18 million new cases of cancer in 2018 (https://gco.iarc.fr/) [1] and radiotherapy (RT) is used to treat 50–60% of cancers [2]. For medical imaging and image-guided interventions, the total exposure in the USA has increased 6-fold since 1980 [3]. However, potential adverse health effects of radiation exposure for patients, as well as for medical staff, especially with a focus on individual differences in radiosensitivity, are poorly understood. 

Radiosensitivity is a measure for the reactions of cells, tissues, or individuals to ionizing radiation (IR). Subjects with increased reactions are described as radiosensitive, when compared to a majority of other “normal” responding individuals [4,5,6]. The reactions include inflammation, fibrosis, cardiovascular illness, cataracts, and cognitive decline [7]. The occurrence and severity varies among individuals and may be affected by genetic as well as by life style factors. In 5–10% of patients the use of RT is limited by the occurrence of acute, clinically diverse, strong radiogenic side effects of normal tissue in the radiation field, leading to suboptimal tumour control or to serious impairment of the quality of life for patients [8,9,10]. A reliable, pre-therapeutic identification of radiosensitive patients would improve therapy because an individual dose adjustment could be applied. Furthermore, the identification of radiosensitive persons would be a valuable step in the protection of occupationally exposed persons. To foster research in this field, two radiation research platforms, Multidisciplinary European Low Dose Initiative (MELODI) and European Alliance Medical Radiation Protection Research (EURAMED), declared individual differences in radiation sensitivity as a key research priority. 

In a small subset of patients the severe reactions can be ascribed to known radiation hypersensitivity syndromes, such as Ataxia–Telangiectasia (A–T), Fanconi anaemia (FA) or Nijmegen Breakage Syndrome (NBS) [11,12,13]. As late as 2010, children with A–T mutations have succumbed to death following RT [14]. These genetic syndromes, however, only comprise about 1% of the patients demonstrating severe side effects [15] and most of the enhanced tissue reactions cannot be explained by known genetic disorders. 

Some further genetic associations were suggested by candidate gene approaches as well as by genome-wide association studies in radiotherapy patients. However, only a small proportion of radiosensitive individuals could be identified [16]. Additionally, functional assays such as DNA double stand break repair, induction of chromosomal aberrations, and radiation-induced apoptosis in ex vivo irradiated blood lymphocytes, have been described as predictors of radiosensitivity [17]. In parallel, a substantial number of IR-induced transcriptional and translational alterations were reported [18]. These studies benefit from recent technical developments in omics applications, which facilitate the cost effective quantification of numerous candidates, including posttranslational modifications of proteins. However, for most of the candidates, the potential correlation between IR-induced deregulation and radiosensitivity is under discussion. 

Therefore, the purpose of this paper is to present the *status quo* of our knowledge of IR-induced changes in protein expression in normal tissue that can be correlated to radiosensitivity. We focus on proteins and protein modifications, as, due to posttranscriptional regulatory processes, the alterations in protein levels may describe the actual cell state, inclusive stress responses, more precisely than transcriptome changes [19]. The future goal will then be to establish protein biomarkers for the identification of radiosensitive or radio-resistant individuals. This will help to personalise treatment strategies to cancer patients during RT or help to assist an individualised risk assessment process by identifying and protecting occupationally radiation-exposed persons.

### 1.2. Objectives 

The aim of this systematic review (SR) is to investigate the IR-induced changes, both in vivo and in vitro, in the human proteome that can be correlated to radiosensitivity. 

## 2. Methods

### 2.1. Protocol and Registration

The review protocol [20] was registered to International Prospective Register of Systematic Reviews (PROSPERO) on 10.11.2020 (CRD42020220064).

### 2.2. Eligibility Criteria

Studies that comply with elements of Population, Exposure, Comparators, and Outcome (PECO) were eligible for this SR. The full description of PECO parameters was provided in the protocol [20]. In short, the population for this SR were primarily humans or human-derived non-tumour tissue and secondary non-tumour cell lines that were exposed to ionizing radiation. This population was compared to non-exposed individuals or *in vitro* cultures. Changes in expression of proteins after the exposure, which were associated with radiosensitivity, were defined as the outcome of this review. Only primary peer-reviewed published studies in English language were considered. As a study on ionizing radiation protein biomarkers for epidemiological studies was published in 2012 [21], studies between 2011 and 2020 were investigated in this SR. 

### 2.3. Information Sources

Studies were identified using electronic databases and with consultations of field experts. The authors of the studies were not contacted for further studies or questions regarding the paper. 

### 2.4. Search 

NCBI PubMed (https://pubmed.ncbi.nlm.nih.gov/) [22] and ISI Web of Knowledge (v.5.34) (https://www.webofknowledge.com/) [23] were used to perform the searches. In addition, papers were also added manually. Search strings included a combination of population, exposure, and outcome elements and the applied search strings for ISI Web of Knowledge are provided in Supplementary Information 1. The Pubmed IDs of identified studies from manual as well as database searches were entered in Microsoft Excel and the duplicates (same studies in different databases) were removed using the built-in “Remove duplicate” tool. 

### 2.5. Study Selection

A two-phase screening was performed by authors Dietz and Subedi in parallel. In phase I screening, title, abstract, and key words of all of the studies were cross-checked manually with the inclusion and exclusion criteria provided in the protocol [20]. The articles that were excluded after phase I screening are provided in Supplementary Information 2. A phase II screening (full-text screening) was performed on the remaining articles after phase I screening. The articles excluded after phase II screening, along with the reasons excluded are also given in Supplementary Information 2. Any disagreements between the reviewers was solved either in consensus, or by involving a third reviewer (Moertl or Gomolka) if necessary. The articles retained after phase II screening were used for Synthesis without Meta-analyses (SWiM).

### 2.6. Data Collection Process

The data collection was performed in Google Sheets by Subedi, Dietz, and Moertl, with one reviewer entering the data and the other person confirming it. The data were finally processed with Microsoft Excel. The form for data extraction is submitted in Supplementary Information 3, along with this review. Any disagreements were solved by consensus or by involving a third reviewer. In the case of missing information, the authors were not contacted and was denoted with ‘nr’. 

We extracted information about: the name of the protein; the fold change ratio after IR; bio fluids or cell lines being investigated; the method used to determine the fold change; the quality and quantity of IR; the characteristics of the donor(s) (age, sex, and diseased or healthy); eligibility criteria of the patients; the method used to quantify radiosensitivity (e.g., viability testing); the replicates performed for the experiment and the statistics to accompany the fold changes; the outcome of the change in protein expression; post-translational modification; and conflict of interest. The findings were summarised and the heterogeneity of the data was compared visually in form of tables. 

### 2.7. Grouping Studies for Synthesis

This SR was performed to investigate the changes in protein expression in normal tissue after exposure to ionizing radiation. Therefore, the in vivo and in vitro studies were grouped together and no differences were made between the different radiation qualities. The doses are provided in Gray (Gy) and the dose-rates are provided in (Gy/min).

### 2.8. Standardised Metric and Transformation Used

The increase or decrease in protein expression after IR (fold changes, Equation (1)) was used as a measure of effect size of the exposure. The fold changes were not calculated in this manuscript but taken from the respective studies.

(1) $$\;Fold\;change\;\left(protein\right)=\;\frac{Protein\;expression\;after\;IR}{Protein\;expression\;before\;IR}$$

### 2.9. Synthesis Methods

For each comparison, the null hypothesis represented by *p*-value, or in certain cases by an adjusted *p*-value resulting from multiple testing, was used as synthesis method for each outcome. 

### 2.10. Certainity of Evidence

Studies which contained commonly deregulated proteins were pooled together. Studies were given an initial confidence rating of high, moderate, low, or very low based on the presence of features (controlled exposure, exposure prior to outcome, individual outcome data, and the use of comparison group). Following the OHAT method, which is based on Grading of Recommendations Assessment, Development and Evaluation (GRADE) working group guidelines, the studies were up- or downgraded. The factors increasing confidence were magnitude of the effect, dose response, residual confounding, and consistency, whereas the factors decreasing confidence were risk of bias, unexplained inconsistency, indirectness, and imprecision. 

## 3. Results 

**Figure 1:**
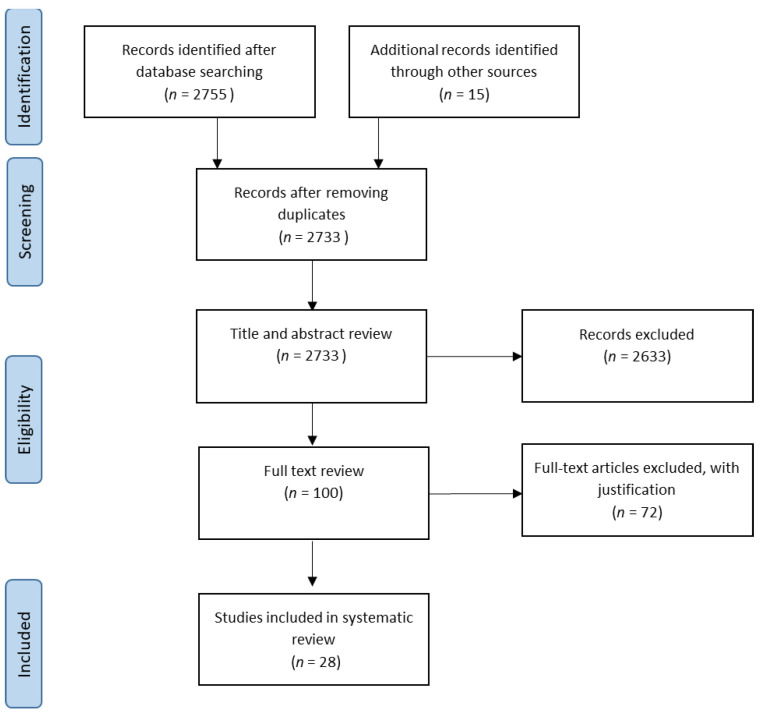
PRISMA flowchart that displays the number of records identified (2733), the number of records screened for a full-text review (100), and the number of records included in the review (28).

After database searching and inclusion of manual sources, 2733 studies were identified. The records were screened for title, abstract, and key words, and 100 articles were selected for a full-text review. Finally, 28 articles were included for this SR (Figure 1). In the included articles, 13 studies examined solely DNA repair foci, 12 studies investigated proteins other than repair foci, with 3 studies also including repair foci. 

### 3.1. Study Characteristics of the included Articles

The 16 studies that used repair foci to determine individual differences in radiosensitivity included 10 cohort studies (van Oorschot et al., 2014 [24], Vasireddy et al., 2010 [25], Bourton et al., 2011 [26], Mumbrekar et al., 2014 [27], Poulilou et al., 2015 [28], Lobachevsky et al., 2016 [29], Buchbinder et al., 2016 [30], Granzotto et al., 2016 [31], Djuzenova et al., 2013 [32], and Goutham et al., 2012 [33]) and 6 model system (Vandersickel et al., 2010 [34], Martin et al., 2014 [35], Martin et al., 2011, [36], Minafra et al., 2015 [37], Miyake et al., 2019 [38], and Nguyen et al., 2019 [39]). The detailed study characteristics of these studies is provided in Table 1a.

Amongst the studies, which investigated proteins other than repair foci, 15 studies were included: five cohort studies (Braicu et al., 2014 [40], Rodruiguez-Gil et al., 2014 [41], Skiöld et al., 2015 [42], Yu et al., 2018 [43], and Lacombe et al., 2019 [44]) and 10 studies on model systems (Cao et al., 2011 [45], Park et al., 2012 [46], Fekete et al., 2015 [47], Minafra et al., 2015 [37], Nishad and Ghosh, 2015 [48], Shimura et al., 2015 [49], Yim et al., 2017 [50], Miyake et al., 2019 [38], Nguyen et al., 2019 [39], Moertl at al., 2020 [51]). In total, 5 of these 10 studies were conducted with peripheral blood mononuclear cells (PBMCs) (Yu et al., 2018, Nguyen et al., 2019, Lacombe et al., 2019, Skiöld et al., 2015, and Nishad and Ghosh, 2015), and one with PBMCs-derived extracellular vesicles (Moertl et al., 2020). The detailed study characteristics are provided in Table 1b.

In total, the 28 included studies identified 76 proteins, which were correlated with normal tissue radiosensitivity. The results were prioritised so that the proteins identified in more than one study, regardless of the direction of regulation, along with their role in radiation response, were described further. Besides changes in repair foci (γH2AX and TP53BP1 quantities), the proteins were identified in more than one study are Vascular endothelial growth factor (VEGF), Caspase 3, p16^INK4A^ (Cyclin-dependent kinase inhibitor 2A, CDKN2A), Interleukin-6, and Interleukin-1B.

### 3.2. IR-Induced Changes in Repair Foci Proteins

H2AX, a variant of the histone protein H2A, is located in the nucleus and its functions include chromatin organisation and DNA damage response. In case of DNA double strand break damage, its phosphorylation by PI3 kinases ATM, ATR, and DNAPKcs signals the damaged site, and recruits downstream DNA repair proteins [52,53,54,55]. The phosphorylated isoform on serine 139 is termed as γH2AX [52,53]. The initial γH2AX signal develops and expands within the first hour after DNA damage induction. With subsequent repair of the damaged sites, the signal decreases again. Depending on the amount and the complexity of the DNA damage and on DNA repair capacity, the differences in DNA repair kinetic and residual foci level are observed [56]. In addition to γH2AX, another component of the DNA double strand break repair machinery, TP53BP1 (Tumour Protein P53 Binding Protein 1) [32,36], was also identified as a target candidate to predict radiation sensitivity. TP53BP1 plays an essential role in the canonical non-homologous end joining (NHEJ) repair of DNA double strand breaks (DSB), which is the main repair pathway of DSB in G0–G1 cell cycle phase, e.g., in peripheral blood lymphocytes [57]. TP53BP1 clusters appears during radiation response and disappears in a similar time dependent kinetic as γH2AX foci do. γH2AX and TP53BP1 quantities were measured by immunofluorescence microscopy in most of the studies except for Bourton et al. and Pouliliou et al. In their studies, γH2AX expression was analysed by fluorescence-activated cell sorting (FACS) and western blot, respectively. The IR-induced alterations of γH2AX and TP53BP1 expressions are presented in detail in Supplementary Information 4. 

In all studies, irradiation was performed with gamma or X-ray radiation at a high dose rate and doses from 0.5 2.0 Gy. Studies were performed in different cell lines (fibroblast, lymphoblastoid, epithelial cell lines) harbouring DNA repair defects, or in primary cells (blood cells, hair follicle) from cancer patients. From all parameters investigated, such as basal foci level, radiation induced foci and residual foci at later repair time points, elevated levels of residual γH2AX or TP53BP1 foci appear to be robust to identify radiosensitive cells or individuals. 

DNA repair deficient individuals demonstrate delayed development of the initial DNA damage or delayed DNA repair, resulting in an increased level of residual damage after 24 hours [35,36]. Therefore γH2AX is considered as a putative predictive biomarker to detect radiation sensitive individuals harbouring DNA repair defects by performing an *in vitro* challenging assay and investigating signal development and disappearance [26,56,58]. Promising studies demonstrating a positive association of increased residual damage in ATM [35,36,59], Ligase IV deficient radiation sensitive individuals [30,36], and in cancer patients experiencing strong acute or late side effects from the radiation treatment [24,25,26,27,28,29,32] are presented. However, the literature overview has shown multiple factors, such as high variability of the assay itself, the lack of a standardized protocol including a fixed in vitro exposure dose, repair time point to analyse residual foci, and comparator group, bias the results. Therefore, correlation to outcome parameters such as genetically defined repair deficient cell lines and patients, as well as association to clinical radiation sensitivity, still remain contradictory [5,34,60]. Our systematic review and others show that although γH2AX and TP53BP1 expressions have the potential to predict an in vitro radiation response in a number of patients; large cohorts need to be analysed by standardised protocols to improve the robustness and sensitivity of the assay, and to decipher the subgroups of patients for which the assay is a meaningful tool to predict detrimental radiation reactions [5,18,61].

### 3.3. IR-Induced Deregulated Proteins Excluding Repair Foci and Risk of Biases

An aim of our SR is to discover new feasible markers on protein levels that are associated with radiosensitivity, besides repair foci proteins. To provide a rich reflection of evidence for the reader, we included both significantly deregulated and not deregulated proteins in Supplementary Information 5. There is comparatively little evidence published on this topic within the inclusion parameters specified (especially the correlation to radiosensitivity). Therefore, if the studies included experiments that depict cell survival, the paper was incorporated to the synthesis, irrespective of a direct correlation of the outcome to radiosensitivity. Table 2 presents the evaluation of studies containing proteins, other than repair foci, on all applicable risk of bias (RoB) questions as developed by the Office of Health Assessment and Translation (OHAT) [62]. The questions concerning the RoB tools and the criteria to judge the different biases are provided in the protocol [20] and in Supplementary Information 6. Although a set of 11 questions was used to evaluate the studies, the studies were categorised into three tiers (T1, T2, or T3) primarily based on the responses to the following key questions (Supplementary Information 7)

1. Can we be confident in the exposure assessment?
2. Can we be confident in the outcome assessment?
3. Did the study design or analyses account for important confounding and modifying variables?

None of the studies were categorised in T1, one study (Park, 2012) was categorised into T3, and the rest were categorised as T2. The RoB questions are suited to cohort and human clinical trials compared to model systems. Concealment, randomisation, and blinding in most studies on model systems are not performed because (i) it is usually a single person that performs the studies and (ii) it is not a common practice to conceal the study groups from the researcher. Therefore, most studies received a ‘probably high risk of bias’ assessment in randomisation, concealment, and blinding domains. Although randomisation is performed during the accessing of outcomes, for example, when performing mass spectrometric analyses or measuring γH2AX quantities on coded slides, more often than not, it is not reported to ensure brevity during publication. Based on the results from this SR, we can recommend that studies on model systems should take care of randomisation, concealing of study groups, blinding the accessors, and, most important, reporting them. 

The proteins that were reported in at least two studies (Table 3) are explained further: 

#### 3.3.1. Vascular Endothelial Growth Factor (VEGF)

VEGF induces endothelial cell proliferation, promotes cell migration, inhibits apoptosis, and induces permeabilization of blood vessels [63,64]. Furthermore VEGF is associated with autophagy, a conserved and essential mechanism for both protecting and killing cells during stress response [65]. Autophagy is carried out by lysosomal degradation of macroproteins or even whole organelles [66,67] and is thought to contribute to normal tissue and tumour radio-resistance [68,69,70]. 

Nguyen et al. reported an increase in VEGF secretion 48 h after exposure to 2 Gy IR (^137^Cs, dose rate 2.7 Gy/min) in CCR6+Th17 T cells, which are highly sensitive to IR-induced senescence. This may contribute to IR-induced normal tissue damage and might facilitate tumour recurrence and metastasis after radiotherapy [39]. Braicu et al. investigated VEGF levels in the serum of patients with locally advanced FIGO stage Ib–IIb cervical cancer before and after chemoradiotherapy (6 MV photon linear acceleration). They demonstrated that a decrease in VEGFA concentration leads to an increase in overall survival; an increase of more than 500 pg/mL VEGF in serum negatively influenced the overall survival due to the resistance to chemoradiotherapy [40]. Fekete et al. described an increase in VEGF levels in non-irradiated MSCs (Bone-Marrow-Derived Mesenchymal Stromal Cells), whereas no significant change was observed in irradiated MSCs (30 Gy, 7, 14, 21, and 28 d post IR with ^137^Cs) [47]. 

VEGF is a key mediator of neovascularisation and is highly expressed in cancer cells and tumour-associated stromal cells [71]. In a meta-analysis conducted to evaluate the relationship between serum VEGF expression and radiosensitivity in Asian non-small cell lung cancer (NSCLC) patients, it was established that lower expression of VEGF led to a longer overall survival and could be a useful biomarker to predict radiosensitivity and prognosis of NSCLC patients [72]. Hu et al. reported IR-induced increased VEGF expression in HeLa cells in vivo and in vitro and a knockdown of VEGF expression in HeLa cells indicated increased cellular sensitivity to radiation [73].

The effect of radiation exposure on VEGF seems to be cell type dependent. However, first in vitro and in vivo studies suggest its importance for normal tissue radiosensitivity. Therefore, it is a promising candidate marker to study radiosensitivity in future projects. 

#### 3.3.2. Caspase 3

Caspase 3 is involved in the activation cascade of several caspases responsible for apoptosis by proteolytically cleaving poly(ADP-ribose) polymerase (PARP). Furthermore it cleaves and activates Caspase-6, -7, and -9 [74].

Both, Cao et al. [45] and Nguyen et al. [39] conducted their studies on ^137^Cs irradiated T cells (dose rate 2.7 and 4.8 Gy/min, respectively) and observed a radiation induced increase in Caspase 3 concentration, where Cao et al. reported a higher increase in radiosensitive CD4+CD25+ Treg cells compared to normo-sensitive CD4+CD25- T cells after overnight incubation post 0.94, 1.875, and 7.5 Gy. Nguyen et al. described a greater Caspase 3 activation (48 h post 2 Gy) in CCR6negTh cells compared to CCR6+Th17 that are rather prone to IR-induced senescence than to apoptosis. When lymphocytes from healthy donors were irradiated with 1, 2, or 4 Gy (^60^Co), a dose-dependent increase in active Caspase 3 was observed that included high intra-individual variability [75]. This suggests that Caspase 3 could effectively be used as a tool to detect individual differences in radiosensitivity, which could be used on patients before they undergo radiotherapy. In a study conducted in MCF-7 breast cancer cells, it was discovered that Caspase 3 plays a critical role in radiotherapy-induced apoptosis, and this suggests that Caspase 3 deficiency may contribute to the radio-resistance of breast cancers [76]. Although an activation of Caspase 3 seems to be a potential candidate to define radiosensitive cells, due to limited numbers of donors (5 and 32), the results needs to be validated in further studies.

#### 3.3.3. p16^INK4A^ (Cyclin-Dependent Kinase Inhibitor 2A, CDKN2A)

p16 acts as a negative regulator of normal cell proliferation by inhibiting CDK 4 and CDK 6 interaction with cyclin D and the phosphorylation of retinoblastoma protein, prohibiting progression from G1 phase to S phase [77,78]. p16 is a known marker for senescence through its contribution to the repression of proliferation-associated genes. High-Mobility Group A proteins act together with p16 to promote senescence-associated heterochromatic foci (SAHF) formation () and proliferative arrest [79]. 

Miyake et al. observed that an increase in p16 expression in keratinocytes (passage 1, 2, and 3), was characterised as radio-resistant but not in fibroblasts or induced pluripotent stem cells (iPSCs) 72 h after 2 Gy ^60^Co γ irradiation (dose rate 2.7 Gy/min) [38]. Nguyen et al. showed that p16 expression was higher in CCR6+Th17 cells (radio-resistant compared to Treg cells) 48 h after 2 Gy ^137^Cs with a dose rate of 2.7 Gy/min IR and led to IR-induced senescence [39]. In contrast, studies have shown that p16 expression leads to radio-sensitisation in cancer cell lines [80,81,82]. Since p16 is known to be a marker for senescence and the study results between tumour cell and normal cells are controversial, p16 is not a promising marker to determine individual differences in radiosensitivity.

#### 3.3.4. Interleukin-6 (IL-6)

The pleiotropic cytokine IL-6 comprises a wide variety of biological functions including immunity, tissue regeneration, and metabolism [83]. It is a potent inducer of the acute phase response and a rapid production of IL-6 contributes to host defence during infection or injury. IL-6 expression is tightly regulated, both transcriptionally and post-transcriptionally and its immoderate production causes severe inflammatory diseases.

Cao et al. reported that IL-6 is significantly downregulated in response to 0.94 and 1.87 Gy (^137^ Cs, dose rate 4.8 Gy/min) in radiosensitive Treg cells, but not in T cells showing a normal sensitivity [45]. The study of Fekete et al. found increased IL-6 levels during culture of both exposed and non-exposed MSCs (bone-marrow-derived mesenchymal stromal cells) 7, 14, 21, and 28 d post IR with ^137^ Cs [47]. 

Chen et al. showed that irradiation-induced IL-6 and the subsequent recruitment of myeloid-derived suppressor cells could be responsible for tumour regrowth [84]. Several clinical observations have documented increased IL-6 levels in plasma from patients with therapy-resistant metastatic disease compared to patients with earlier stages of the disease and healthy individuals. Higher levels of IL-6 in body fluids were associated with poor prognosis and survival [85,86,87,88,89,90]. These findings fit to the results of Cao et al. showing that downregulation of IL-6 enhances radiosensitivity. Concerning normal tissue, more evidence is needed to confirm these findings.

#### 3.3.5. Interleukin-1 Beta (IL-1β)

IL-1β is a proinflammatory cytokine and works in coaction with interleukin-12 and induces interferon gamma synthesis from T-helper 1 cells [91]. By inducing VEGF production synergistically with TNF and IL-6, IL-1β is involved in angiogenesis [92]. 

Like for IL-6, Cao et al found a significantly downregulated IL-1β in response to 0.94 and 1.87 Gy ^137^Cs irradiation, delivered with a dose rate of 4.8 Gy/min in radiosensitive Treg cells, but not in normal sensitive T cells [45]. Secretion of IL-1β was increased only in CCR6negTh and not in CCR6+Th17 cells 48 h after 2 Gy (^137^Cs, dose rate 2.7 Gy/min) irradiation according to Nguyen et al. [39]. Chen et al. reported a significant overexpression of IL-1 beta in cancer specimens compared to non-malignant tissues. By blocking IL-1 β, tumour growth, invasion ability, and treatment resistance were attenuated [93]. Regarding the diverse observations of Cao et al. and Nguyen et al., IL-1β does not seem to be a favourable biomarker.

The studies that contained the previous markers were further evaluated based on a Grading of Recommendations Assessment, Development and Evaluation (GRADE) approach (Table 4). Each study received an initial confidence rating based on the presence or absence of four features, which were (1) controlled exposure, (2) exposure prior to outcome, (3) individual outcome data, and (4) use of comparison group. The studies that received the same initial confidence were pooled together and either up-graded depending on magnitude effect, dose response, residual confounding, consistency, or downgraded based on risk of bias, unexplained inconsistency indirectness, or imprecision. The factors that decreased confidence were risk of bias, unexplained consistency, and indirectness. The detailed information is provided in the protocol [20].

**Figure 2:**
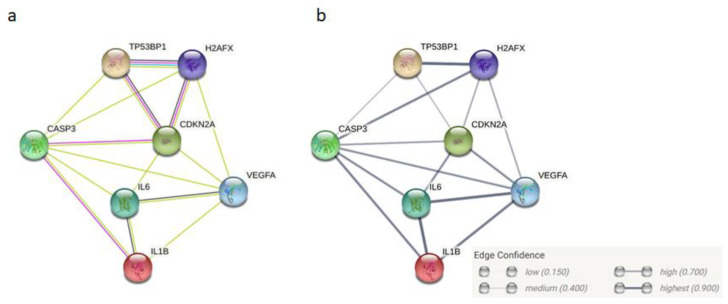
Protein-protein interaction enrichment network generated in STRING 11.0 using proteins identified in at least two studies: p16 (CDKN2A), VEGFA, IL6, IL-1β, CASP3, TP53BP1, and γH2AX: (**a**) The lines represent protein–protein association where pink lines are known experimentally determined interactions, blue from curated databases, green are from text-mining, and black represents co-expression; (**b**) The thickness of the edges display the confidence in interaction: medium (0.400), high (0.700), and highest (0.900) in this network.

Significant interactions for aforementioned proteins, TP53BP1, and γH2AX (Figure 2), were identified when an in silico protein enrichment was performed on the STRING 11 database [94,95]. The generated network consisted of 7 nodes that are connected via 15 edges, whereas only 7 edges would be expected when using only 7 proteins for analysis. The interactions suggest that the proteins are likely to be biologically connected. 

## 4. Outlook 

First of all, it is important to understand the proteomic landscape of normal tissues. Different tissues and cell types harbour divergent baseline protein expression [96]. Most of the studies are focused on blood or blood cell-derived changes, but normal tissue reaction post IR is multifaceted and dependent on tissue types. Therefore more mechanistic studies are required to identify the tissue-specific impact of proteins on radiosensitivity. In this regard the validation of proteins for different dose rates will be an important point in future studies, because new developments in radiotherapy, such as ultra-high dose radiotherapy (FLASH) use much higher dose rates which may affect radiosensitivity differentially.

Second, radiosensitivity is a complex issue as many risk factors modify the radiation reaction, thus determining each predictor’s overall impact is difficult to characterise. Some of the factors that influence radiosensitivity and complicate the discovery of a ubiquitous applicable biomarker are specified in this section. 

There are several known hereditary hyper-radiosensitive disorders arising from rare mutations in DNA repair genes of large effect. All belong to XCIND syndromes, named after distinct hypersensitivity to ionizing radiation (X-ray), cancer susceptibility, immunodeficiency, neurological abnormality, and double-strand DNA breakage. Examples of such syndromes are Ataxia telangiectasia, Fanconi anemia, Ligase IV syndrome, Radiosensitive severe combined immunodeficiency disease (RS-SCID), Radiosensitivity, immunodeficiency, dysmorphic features, and learning difficulties (RIDDLE) syndrome, or ataxia telangiectasia and Rad3-related protein (ATR)-Seckel syndrome [15,97,98,99]. Polymorphic variants, as well as mutations in multiple genes that lead to similar or different DNA damage response pathways, will contribute to genetically defined radiosensitivity in a complex manner. 

Age and gender are crucial factors influencing individual differences in radiosensitivity. Children aged 0–5 years are expected to be the most sensitive group concerning radiation-induced leukaemia, as well as skin, breast, thyroid, and brain cancer for both high and low dose radiation exposures [100,101,102,103,104,105]. Sex influences the radiation response and the radiation-induced cancer risk [106]. Epidemiological studies from the Chernobyl disaster in 1986 and the Hiroshima and Nagasaki atomic bomb survivors provide evidence that females possess a greater risk for solid cancers [107,108,109] mainly due to cancer of reproductive tissue [110] and thyroid and brain cancer [106,111].

The anatomical structure (organ size, body mass index), as well as breathing rates, and individual metabolism of exposed individuals alter radiation doses received by organs and tissues, which leads to inter-individual variations [112,113,114,115,116]. Lifestyle is another aspect that affects individual cancer susceptibility when radiation exposure is considered. Although smoking and ionizing radiation exposure are the most studied influences, other co-exposures such as heavy metals, medication, alcohol consumption, dietary habits, and combined exposure to other radiation qualities such as radon needs to be taken into account [117,118,119]. Additionally, already diseased individuals cope poorly to radiation exposure compared to healthy ones. [120,121]. 

## 5. Conclusions 

The fact that there is a clear evidence that not all individuals share the same radiation-induced risk of adverse health outcomes is also backed by the reports from the advisory group on ionizing radiation (UK) [122] and International Commission on Radiological Protection (ICRP) [123]. Radiosensitivity represents a complex phenotype and this is perhaps why we identified few IR-induced proteins (γH2AX, TP53BP1, VEGF, CASP3, CDKN2A, IL-6, and IL-1B), that correlated to radiosensitivity, when common markers in at least two studies were considered. These candidate proteins and their possible interaction partners should be investigated further, to discover biomarkers that can properly define radiation sensitivity.

The need to discover biomarkers for disease risk or susceptibility of radiation related risks for individuals or population subgroups is vital and also stressed by MELODI platform [124]. Not only would patients benefit by an individualised cancer treatment but also individualised risk assessment and prevention measurements can protect at-risk occupationally exposed individuals more efficiently. This systematic review highlights the fact that there is a lack of basic studies with a focus on normal tissue in contrast to tumour tissues. More studies based on functional assays are needed to survey the role of specific proteins in different normal tissues. In addition, the frequently statistically underpowered studies do strengthen the need to use large cohorts, as well as very sensitive methods for the biomarker search, as well as focusing on functional tests of potential markers in different accessible normal tissue (lymphocytes, fibroblasts, keratinocytes, and body fluids).

## 6. Differences between Protocol and the Review

The GRADE tool to up- or downgrade studies was not performed on all studies but only on studies that included proteins, other than repair foci, reported in at least two studies.

## Figures and Tables

**Table 1:** (a) Study characteristics of included records concerning repair foci. (b) Study characteristics of included records containing proteins other than repair foci.

(a)								
Author, Date	Title	Study Design	Sample-Size	Methods Used	Statistical Method	Repair Foci	Viability	Cell System
Vasireddy, 2010 [25]	H2AX phosphorylation screen of cells from radiosensitive cancer patients reveals a novel DNA double-strand break repair cellular phenotype	Cohort	29	IF	nr	γH2AX	(RTOG)	lymphoblastoid cell lines (LCLs)
Bourton, 2011 [26]	Prolonged expression of the γ-H2AX DNA repair biomarker correlates with excess acute and chronic toxicity from radiotherapy treatment	Cohort	30	FACS	unpaired t-test	γH2AX	(RTOG)	lymphocytes
Goutham, 2012 [33]	DNA double-strand break analysis by γ-H2AX foci: a useful method for determining the overreactors to radiation-induced acute reactions among head-and-neck cancer patients	Cohort	54	IF	nr	γH2AX	(RTOG)	lymphocytes
Djuzenova, 2013 [32]	Radiosensitivity in breast cancer assessed by the histone γ-H2AX and 53BP1 foci	Cohort	69	IF	Student’s t-test or one way ANOVA	γH2AX, 53BP1	(RTOG)	PBMCs
Mumbrekar, 2013 [27]	Influence of double-strand break repair on radiation therapy-induced acute skin reactions in breast cancer patients	Cohort	118	IF	t test, ANOVA followed by Tukey multiple comparison tests and Pearson correlation test	γH2AX	(RTOG)	lymphocytes
Oorschot, 2013 [24]	Reduced Activity of Double-Strand Break Repair Genes in Prostate Cancer Patients With Late Normal Tissue Radiation Toxicity	Cohort	61	IF	Continuous variables: Shapiro-Wilk test, normal distributed data: unpaired Student t test, non-normal distributed data: Mann–Whitney test	γH2AX	Late toxicity using EORTC	lymphocytes
Granzotto, 2015 [31]	Influence of Nucleoshuttling of the ATM Protein in the Healthy Tissues Response to Radiation Therapy: Toward a Molecular Classification of Human Radiosensitivity	Cohort	117	IF	ANOVA	γH2AX, pATM	Common Terminology CTCAE, RTOG	fibroblasts
Pouliliou, 2015 [28]	Survival Fraction at 2 Gy and γH2AX Expression Kinetics in Peripheral Blood Lymphocytes From Cancer Patients: Relationship With Acute Radiation-Induced Toxicities	Cohort	89	WB	nr	γH2AX	Common Terminology CTCAE, Trypan Blue assay	PBMCs
Lobachevsky, 2016 [29]	Compromised DNA repair as a basis for identification of cancer radiotherapy patients with extreme radiosensitivity	Cohort	28	IF	Unpaired t-test, Mann–Whitney test	γH2AX	(RTOG)	lymphocytes, hair follicles
Buchbinder, 2018 [30]	Application of a radiosensitivity flow assay in a patient with DNA ligase 4 deficiency	Cohort	11	IF	nr	γH2AX	known sensitivity LIG4-SCID	T cells
Vandersickel, 2010 [34]	Early Increase in Radiation-induced γH2AX Foci in a HumanKu70/80 Knockdown Cell Line Characterised by an Enhanced Radiosensitivity	model system	1	IF	nr	γH2AX	known sensitivity Ku70i	LVTHM cells synchronised in the G0–G1 phase, Ku70i cells synchronised in the G0–G1 phase
Martin, 2011 [36]	Assessing ’radiosensitivity’ with kinetic profiles of γ-H2AX, 53BP1 and BRCA1 foci	model system	15	IF	unpaired t test	γH2AX, 53BP1	clonogenic survival	LCL
Martin, 2014 [35]	Homozygous mutation of MTPAP causes cellular radiosensitivity and persistent DNA double-strand breaks	model system	4	IF	Student’s t-test	γH2AX	clonogenic survival	LCL
Minafra, 2015 [37]	Gene Expression Profiling of MCF10A Breast Epithelial Cells Exposed to IOERT	model system	1	IF	nr	γH2AX	clonogenic survival	MCF10A
Miyake, 2019 [38]	DNA Damage Response After Ionizing Radiation Exposure in Skin Keratinocytes Derived from Human-Induced Pluripotent Stem Cells	model system	1	IF	Student’s t test (1-tailed)	γH2AX, 53BP1	Cell survival WST-8 assay; TUNEL assay	Normal human skin fibroblast NB1RGB, iPSCs NB1RGB C2, NB1RGB KCs 1stP, NB1RGB KCs 2ndP, NB1RGB KCs 3rdP
Nguyen, 2019 [39]	Human CCR6+ Th17 Lymphocytes Are Highly Sensitive to Radiation-Induced Senescence and Are a Potential Target for Prevention of Radiation-Induced Toxicity	model system	32	IF	two-tailed Mann–Whitney U-test, Kruskal–Wallis test	γH2AX	Annexin V-FITC; Senescence-associated β-Galactosidase	Treg, CCR6+Th17, CCR6negTh
**(b)**								
**Author, Date**	**Title**	**Study Design**	**Sample-Size**	**Methods Used**	**Statistical Method**	**Results (Protein Name)**	**Viability**	**Cell System**
Braicu, 2014 [40]	Role of serum VEGFA, TIMP2, MMP2, and MMP9 in Monitoring Response to Adjuvant Radiochemotherapy in Patients with Primary Cervical Cancer – Results of a Companion Protocol of the Randomised NOGGO-AGO Phase III Clinical Trial	Cohort	72	ELISA	Fisher’s exact test	VEGFA, TIMP2, MMP2, MMP9	overall survival	Serum
Rodriguez-Gil, 2014 [41]	Inflammatory Biomarker C-Reactive Protein and Radiotherapy-Induced Early Adverse Skin Reactions in Patients with Breast Cancer	Cohort	159	ELISA	two-sided Student’s t-test	C-reactive protein (CRP)	EASR	plasma
Skiöld, 2014 [42]	Unique proteomic signature for radiation sensitive patients; a comparative study between normo-sensitive and radiation sensitive breast cancer patients	Cohort	17	LC-MS/MS	Student’s t-test	8-oxo-dG, BLVRB, PRDX2, SOD1, CA1, PARK7, SH3BGRL3	RTOG	blood/leukocytes (RTOG 0), blood/leukocytes (RTOG 4)
Yu, 2018 [43]	Cofilin-2 Acts as a Marker for Predicting Radiotherapy Response and Is a Potential Therapeutic Target in Nasopharyngeal Carcinoma	Cohort	70	ELISA	Wilcoxon rank-sum test, t test or one-way analysis of variance (ANOVA)	Cofilin-2	Patients were divided into radiosensitivity and radio-resistance groups according to therapeutic effects	Serum
Lacombe, 2019 [44]	Quantitative proteomic analysis reveals AK2 as potential biomarker for late normal tissue radiotoxicity	Cohort	5	WB	Mann–Whitney test	adenylate kinase 2 (AK2), annexin A1 (ANXA1), isocitrate dehydrogenase 2 (IDH2), HSPA8, Nox4	RILA	T lymphocytes (Grade > 2 breast fibrosis+), T lymphocytes (Grade < 2 breast fibrosis+)
Cao, 2011 [45]	Different radiosensitivity of CD4+CD25+ regulatory T cells and effector T cells to low dose gamma irradiation in vitro	model system	5	FACS, Luminex	Wilcoxon’s signed rank test	Caspase 3, Bax, IL-1 Beta, IL-2, IL-4, IL-6, IL-10, Interferon Gamma, TNF alpha	Annexin V-FITC	CD4+CD25+ regulatory T cells and effector T cells
Park, 2012 [46]	Radio-sensitivities and angiogenic signaling pathways of irradiated normal endothelial cells derived from diverse human organs	model system	1	ELISA	Student’s t-test	angiostatin	clonogenic survival	HHSEC, HDMEC
Fekete, 2015 [47]	Effect of High-Dose Irradiation on Human Bone-Marrow-Derived Mesenchymal Stromal Cells	model system	nr	Luminex	unpaired, two sided Student’s t-test	PDGF-AA, PDGF-AB/BB, GRO, IL-6, VEGF	CyQUANT Cell Proliferation Assay, Trypan blue staining, colony formation	MSCs
Minafra, 2015 [37]	Gene Expression Profiling of MCF10A Breast Epithelial Cells Exposed to IOERT	model system	1	WB	nr	PARP, FAS, Pro-Caspase 8, PLK1, P53, p-EGFR, EGFR, c-MYC,	clonogenic survival	MCF10A cell line
Nishad, 2015 [48]	Dynamic changes in the proteome of human peripheral blood mononuclear cells with low dose ionizing radiation Radiotherapy-Induced Early Adverse Skin Reactions in	model system	8	2DE-MS, WB	Student’s t-test	GRP78, HSP90, PDIA3, PRDX6	trypan blue, PI Staining, alkaline comet assay	PBMCs
Shimura, 2015 [49]	Nuclear accumulation of cyclin D1 following long-term fractionated exposures to low-dose ionizing radiation in normal human diploid cells	model system	1	WB	Student’s t-test	cyclin D1	cell growth assay	WI-38 (detergent insoluble fraction)
Yim, 2017 [50]	Phosphoprotein profiles of candidate markers for early cellular responses to low-dose γ-radiation in normal human fibroblast cells	model system	1	WB, antibody microarray	Student’s t-test	Phospho-Gab2 (Tyr643), Phospho-P95/NBS (Ser343), Phospho-BTK (Tyr550), Phospho-Elk1 (Ser383), Phospho-ETK (Tyr40), Phospho-CaMK4 (Thr196/200), Phospho-MEK1 (Thr298), Phospho-PLCG1 (Tyr1253), Phospho-IRS-1 (Ser612), Phospho-TFII-I (Tyr248), Phospho-IKK-alpha/beta (Ser176/177), Phospho-MEK1 (Thr286), Phospho-Pyk2 (Tyr580), Phospho-Keratin 8 (Ser431), Phospho-ERK3 (Ser189), Phospho-Chk1 (Ser296), Phospho-CBL (Tyr700), Phospho-BTK (Tyr550), Phospho-LIMK1/2 (Thr508/505), p-BTK(Tyr550)/BTK, p-Gab2(Tyr643)/Gab2, p-BTK(Tyr550)/BTK, p-Gab2(Tyr643)/Gab2,	MTT	MRC5, NHDF
Miyake, 2019 [38]	DNA Damage Response After Ionizing Radiation Exposure in Skin Keratinocytes Derived from Human-Induced Pluripotent Stem Cells	model system	1	IF	Student’s t test (1-tailed)	p16	Cell survival WST-8 assay, TUNEL assay	Skin keratinocytes were derived from iPSCs
Nguyen, 2019 [39]	Human CCR6+Th17 lymphocytes are highly sensitive to radiation-induced senescence and are a potential target for prevention of radiation-induced toxicity	model system	32	IF, Luminex	two-tailed Mann–Whitney U-test, Kruskal–Wallis test	Caspase 3, p16Ink4a, p21Cdkn1a, IL-1 Beta, VEGF-A, IL-8, H2A.J	Annexin V-FITC, Senescence-associated β-Galactosidase	CCR6+Th17 lymphocytes
Moertl, 2020 [51]	Radiation Exposure of Peripheral Mononuclear Blood Cells Alters the Composition and Function of Secreted Extracellular Vesicles	model system	5	LC-MS/MS	two-sided Student’s t-test	hemopexin (HPX), syntaxin-binding protein 3 (STXBP3), proteasome subunit alpha type-6 (PSMA6)	sub-G1 fraction, Caspase 3 activity	PBMC-derived EVs

Abbreviations: (a) Criteria for adverse events (CTCAE), European organisation for research and treatment of cancer (EORTC), Fluorescence-activated cell sorting (FACS), Immunofluorescence (IF), not reported (nr), Peripheral blood mononuclear cell (PBMC), Radiation therapy oncology group (RTOG), Western Blot WB). (b) Two-dimensional gel electrophoresis (2-DE), Enzyme-linked immunosorbent assay (ELISA), Immunofluorescence (IF), Liquid chromatography (LC), Mass spectrometry (MS), not reported (nr), radiation-induced lymphocyte apoptosis (RILA), Radiation therapy oncology group (RTOG), Western blot (WB).

**Table 2:** Accessing risk of bias for studies that included proteins other than repair foci.

	Risk of Bias Domains and Ratings	Miyake, 2019	Cao, 2011	Nguyen, 2019	Minafra, 2015	Yim, 2017	Braicu, 2014	Park, 2012	Fekete, 2015	Shumura, 2015	Lacombe, 2019	Skiöld, 2014	Nishad, 2015	Rodruiguez-Gil, 2014	Moertl, 2020	Granzotto, 2016	Yu, 2018
Key criteria	Can we be confident in the exposure characterisation?	--	+	+	++	++	--	--	-	+	-	+	+	-	++	+	--
	Can we be confident in the outcome assessment?	-	--	-	-	+	+	-	+	+	++	+	+	++	-	+	++
	Did the study design or analyses account for important confounding and modifying variables?	+	-	++	+	++	--	-	+	-	+	-	-	++	-	-	+
Other RoB criterion	Was administered dose or exposure level adequately randomised?	-	-	-	-	-	-	-	-	-	--	-	+	-	-	-	+
	Was allocation to study groups adequately concealed?	-	-	-	-	-	-	-	-	-	-	-	-	-	-	-	-
	Did selection of study participants result in appropriate comparison groups?	++	++	-	++	++	++	+	+	-	+	-	+	++	++	-	+
	Were experimental conditions identical across study groups?	-	++	-	-	-		-	-	--	-	+	++	-	++	-	++
	Were the research personnel and human subjects blinded to the study group in the study?	-	-	-	-	-	-	-	-	-	-	-	-	-	-	-	-
	Were outcome data complete without attrition or exclusion from analysis?	-	+	+	++	-	++	+	++	++	++	++	++	++	++	-	++
	Were all the measured outcomes reported?	+	++	++	++	++	++	++	++	-	++	++	++	++	++	++	++
	Were there no other potential threat to internal validity (e.g., statistical methods were appropriate and researchers adhered to study protocol?	--	-	++	-	+	-	-	++	+	++	-	+	+	++	+	++
Final Category		T2	T2	T2	T2	T2	T2	T3	T2	T2	T2	T2	T2	T2	T2	T2	T2

Definitely high risk of bias (--), probably high risk of bias (-), probably low risk of bias (+), definitely low risk of bias (++).

**Table 3:** List of proteins identified in at least two studies, not concerning repair foci.

Priority Group	Author, Date	Marker	Outcome	Cell System
1	Braicu, 2014	VEGFA	decrease in VEGFA concentration leads to increase in survival, >500 pg/mL negative influence on survival	Serum
	Fekete, 2015	VEGFA	nr	MSCs
	Nguyen, 2019	VEGFA	resistant compared to Treg	CCR6 + Th17
2	Cao, 2011	Caspase 3 ^#^	radiosensitive	CD4 + CD25 + Treg cells
	Nguyen, 2019	Caspase 3	resistant compared to Treg	CCR6 + Th17
2	Miyake, 2019	p16	resistant compared to primary fibroblasts (IR-induced senescence)	NB1RGB KCs 1stP, 2ndP and 3rdP
	Nguyen, 2019	p16	resistant compared to Treg (IR-induced senescence)	CCR6 + Th17
2	Cao, 2011	IL-6	radiosensitive	CD4 + CD25 + Treg cells
	Fekete, 2015	IL-6	nr	MSCs
2	Cao, 2011	IL-1Beta	radiosensitive	CD4 + CD25 + Treg cells
	Nguyen, 2019	IL-1Beta	sensitive compared to CCR6 + Th17	CCR6negTh

^#^ higher increase in sensitive cells, proteins upregulated post-IR shown in orange, downregulation in blue, and no change in black.

**Table 4:** Accessing confidence in body of evidence in selected studies.

Author, Date	Initial Confidence by Features of Study Design				Initial Confidence Rating	Factors Decreasing Confidence	Factors Increasing Confidence	Final Confidence
	Controlled Exposure	Exposure Prior to Outcome	Individual Outcome Data	Use of Comparison Groups				
Fekete, 2015	×	√	×	×	Very low	Risk of bias Unexplained consistency Indirectness Imprecision	Magnitude effect Dose Response Residual Confounding Consistency	Very low
Braicu, 2014	×	√	√	√	Moderate			Low
Nguyen, 2019	√	√	√	×	Moderate			Moderate
Cao, 2011	√	√	√	√	High			High
Miyake, 2019	×	√	√	×	Low			Low

## Data Availability

Data is provided in the manuscript.

## Supplementary Materials

The following are available online at https://www.mdpi.com/2075-4426/11/2/140/s1: Supplementary Information 1 (Search algorithm for ISI Web of Science), Supplementary Information 2 (List of rejected articles), Supplementary Information 3 (Data extraction sheet), Supplementary Information 4 (IR-induced changes in repair foci proteins), Supplementary Information 5 (IR-induced changes in non-repair foci proteins), Supplementary Information 6 (Risk of bias questions), Supplementary Information 7 (Risk of bias categorisation into T1, T2, and T3, PRISMA and SWiM checklist.
