# Supplementary material for: Ionizing Radiation Protein Biomarkers in Normal Tissue and Their Correlation to Radiosensitivity: A Systematic Review
Source: J Pers Med. 2021 Feb 19;11(2):140. doi: 10.3390/jpm11020140 (PMC7922485; doi:10.3390/jpm11020140)
Supplement: Supplementary file 1 [file jpm-11-00140-s001.zip › Supp Info 3_Data extraction sheet.docx]

Data extraction form

- Study ID (Author, Date)
- Protein marker
- Eligibility criteria (in case of human studies)
- Abundance ratio of the particular protein compared to non-exposed population
- Method used to calculate the abundance ratio
- System of the study (bio fluids/ cell lines)
- Donor of the system (healthy or diseased patients)
- Age, sex, and number of donors
- Replicates for the study
- Radiotherapy or radio chemotherapy
- Ionization parameter (source, company, dose-rate, total dose and if applicable split dose)
- Ionization for controls (0 Gy or sham-irradiation)
- Time after exposure
- Name of viability assay performed
- Post-translational modifications
- Reactive oxygen species
- Statistical method used
- Outcomes
- Conflict of interest
