# Supplementary material for: Ionizing Radiation Protein Biomarkers in Normal Tissue and Their Correlation to Radiosensitivity: A Systematic Review
Source: J Pers Med. 2021 Feb 19;11(2):140. doi: 10.3390/jpm11020140 (PMC7922485; doi:10.3390/jpm11020140)
Supplement: Supplementary file 1 [file jpm-11-00140-s001.zip › Supp Info 7_Risk of Bias questions_Explained.docx]

Internal quality control of selected studies were performed as per risk of bias for human and animal studies from OHAT (https://ntp.niehs.nih.gov/ntp/ohat/pubs/riskofbiastool_508.pdf). The different studies that are taken into consideration are human controlled trials (HCT), cohort (Co), Case-control (CaCo), Cross-Sectional (CrSe), Case series (CaS). RoB tool for *in vitro* experiments, for example, cell culture will be adapted as experimental animal (EA). A summary of how each of the 11 questions receives one of the following four answers (definitely low risk, probably low risk, probably high risk, and definitely high risk) is provided in this document. For an extensive explanation, the readers should refer to the OHAT handbook.

Definitely low risk of bias (++): There is a direct mention of low risk-of-bias practices.
Probably low risk of bias (+): There is not a direct mention, but it can be inferred that low risk-of-bias practice was followed.
Probably high risk of bias (-): There exists an indirect evidence of high risk-of-bias practice, or relevant information about the particular risk-of-bias is not reported.
Definitely high risk of bias (--): Authors explicitly mention a high-risk-of bias practice.

**Selection Bias**

1. Was administered dose or exposure level adequately randomised? (applies to HCT, EA)
   The subjects or *in vitro* probes should have an equal chance of being assigned to control or study groups.

Definitely low risk of bias (++)

There is a direct evidence that human subjects or *in vitro* probes have been randomly allocated so that each subject has equal chance of being assigned to any study group.

Accepted methods for randomization could be using a computer random number generator, coin tossing, referring to a random number table, shuffling cards, or throwing of dice.

Probably low risk of bias (+)

There is an indirect evidence that subjects or *in vitro* probes were randomly allocated to study groups. For example, the authors mention that randomization was performed, but fail to mention the method they use.

Probably high risk of bias (-)

Authors do not report if the randomization was performed, or there is an indirect evidence that a non-random allocation was performed. Non-random allocation could include methods subjects enrolled based on their date of birth, or an investigator-selection of *in vitro* probe from a case number.

Definitely high risk of bias (--)

There is a direct evidence that the subjects and the *in vitro* probes were selected based on a non-random component including judgement of clinician, or preference of participant, results of another test, or the availability of the intervention.

1. Was allocation of study groups appropriately concealed? (HCT, EA)

Proper allocation along with randomization (question 1) is performed so that the exposure level is not given selectively based on potential differences in humans or *in vitro* probes. It states that research personnel do not have an idea beforehand the exposure level that is going to be assigned start of the study. However, in case of patients that needing surgery along with radiation treatment, it is impossible a concealment of the study groups be performed.

Definitely high risk of bias (++)
There is a direct evidence that the research personnel and subjects did not know what study groups subjects were allotted to. Acceptable methods include central allocation, including telephone, web-based and pharmacy-controlled randomization and sequentially numbered opaque or sealed envelopes.

In case of *in vitro* probes, there is a direct evidence that cultivation of probes and treatment with IR is performed by two separate personnel so one they have no chance of knowing the allocation of study groups.

Probably high risk of bias (+)
There is an indirect evidence that the research personnel and the subjects did not know what study groups subjects were allocated.

Or if is certain that adequate allocation concealment would not appreciably bias the results.

Probably low risk of bias (-)
There is an indirect evidence that a possibility existed where the research personnel knew what study groups subjects were allocated to. Methods that lead to a risk of bias include use of unsealed or non-opaque envelopes.

Definitely high risk of bias (--)
There is a direct evidence that it was possible for research personnel and the subjects to know what study groups subjects were allocated to.

In case of *in vitro* probes, the research personnel already knew at the start of experiments which probes would be used as control, and which ones would be treated with IR.

1. Were the comparison groups appropriate? (Co, CaCo, CrSe)

The exposed and non-exposed population must come from similar demographics, including age- and sex-matching. Although OHAT handbook mentions that it applies for cohorts, Case-controls and cross-sectional studies, we have also adapted the question for controlled trials and *in vitro* studies as well.

Definitely low risk of bias (++)

There is a direct evidence that both exposed and non-exposed subjects were similar (e.g., they were recruited from the same eligible population, recruited within the same time frame using same inclusion and exclusion criteria, the subjects were age- and sex- matched, had similar diet and/or smoking/drinking habits).

In case of *in vitro* studies, there is a direct evidence of exposed and non-exposed probes being from the same passage. There is also a direct evidence that the cell lines behave similarly in terms of radiosensitivity.

Probably low risk of bias (+)

There is an indirect evidence that both the exposed and the non-exposed subjects were similar or the differences between the groups would not cause a significant bias

Probably high risk of bias (-)
There is an indirect evidence that the subjects chosen were not similar (e.g., recruited at different time frames, dissimilar demographics, and no age- and sex-matching was performed).

There is an indirect evidence that different passages were used for *in* vitro studies.

Definitely high risk of bias (--)

There is a direct evidence that subjects chosen were not similar (e.g., recruited at different time frames and/or from different demographics)

**Confounding bias**

There is a chance the findings of the study be interpreted falsely if systematic differences in baseline characteristics, prognostic variables, or co-occurring exposures are not taken into consideration. OHAT handbook mentions that this bias applies to cohorts, case-series, cross-sectional, and case series. For human controlled trials and experimental animal studies, this section is covered by the ‘potential threats to internal validity. We have adapted the confounding bias to controlled trials and experimental animal studies.

1. Did the study design or analyses account for important confounding and modifying variables?

Definitely low risk of bias (++)

There is a direct evidence that appropriate adjustments were performed for covariates and confounders, including adjustments in multivariate model.

There is a direct evidence that adjustments were performed if subjects and/or *in vitro* probes were treated with medications along with IR.

There is a direct evidence that adjustment has been performed for research specific bias.

Probably low risk of bias (+)
There is an indirect evidence that appropriate adjustments were performed for covariates and confounders.

Probably high risk of bias (-)

There is an indirect evidence that confounder effects between groups have not been properly adjusted or not accessed using valid and reliable methods.

Definitely high risk of bias (--)

There is a direct evidence that known confounders varied across groups, confounding was demonstrated but not adjusted for final analyses.

**Performance Bias**
Performance bias relates to systemic differences in the care that is given to the study subjects. For ionizing radiation, one of the important experimental procedure is sham irradiation, where the control probes are handled exactly the same, including the transport from laboratories for cell-culture to the chamber for ionizing radiation. This is performed even if the controls are not irradiated.

1. Were experimental conditions identical across study groups? (EA)

Definitely low risk of bias (++)

There is a direct evidence that the control and subject *in vitro* probes were incubated in same conditions and the controls were sham-irradiated as well.

There is also a direct evidence that irradiation for all the subjects was performed with the same machine.

Probably low risk of bias (+)
There is an indirect evidence that the control and the subject *in vitro* probes were incubated in same conditions and the controls were sham-irradiated*.*

There is also an indirect evidence that irradiation for all the subjects was performed with the same machine.

Probably high risk of bias (-)
There is an indirect evidence that the controls and subjects were incubated in different environments and the controls were not sham-irradiated i.e. authors mention 0 Gy.

There is an indirect evidence that irradiation was performed with more than one machine.

Definitely high risk of bias (--)
There is a direct evidence that controls and subjects were incubated in different environments and the controls were not sham-irradiated.

There is also a direct evidence that that irradiation for the subjects was performed with more than one machine.

1. Were the research personnel and human subjects blinded to the group the study? (HCT, EA)

It is imperative that neither the research personnel nor the subjects know which administered dose will be given. This is not possible for cases such as when patients need to undergo surgery.

Definitely low risk of bias (++)
There is a direct evidence that it was impossible for the research personnel to know what exposure level of the subjects of the *in vitro* probes. Methods do achieve blinding could be central allocation, sequentially numbered opaque or sealed envelopes for HCT studies.

For *in vitro* probes, if there is a direct mention that handling of the probes and exposure were performed by separate personnel.

Probably low risk of bias (+)

There is an indirect evidence that research personnel were blinded to the exposure levels of the subjects or *in vitro* probes

Probably high risk of bias (-)
There is an indirect evidence that research personnel were not blinded to exposure levels of subjects or *in vitro* probes

Definitely high risk of bias (--)
There is an direct evidence that research personnel were not blinded to exposure levels of subjects or *in vitro* probes

**Attrition/Exclusion bias**

Attrition or exclusion bias is present when there is a loss or exclusion of study participants or *in vitro* probes.

1. Were outcome data complete without attrition or exclusion bias? (HCT, EA, Co, CaCo, Crse)

Definitely low risk of bias (++)
There is a direct evidence of no loss of subjects during the study. Some participants might opt out during the study for many reasons. If fewer than 10 % of the subjects are missing, and the reason is not related to outcome, the study is treated as having a definitely low risk of bias.

There is a direct evidence in of *in vitro* studies that the cell survival data was monitored throughout the study (e.g., pictures of the probes to study morphology of the cells during the study)

Probably low risk of bias (+)
There is an indirect evidence of no loss of subjects during the study or fewer than 20 % of participants are lost, the reasons for which are unlikely to relate to the outcome of the study.

The authors mention that they checked the cell morphology and survival data during the study but fail to provide any pictures.

Probably high risk of bias (-)

There is an indirect evidence of more than 20 % loss of subjects, for which the reasons are not properly addressed.

Definitely high risk of bias (--)

There is a direct evidence that there is more than 20% loss of subjects and inadequately addressed. There is a direct evidence of imbalance of numbers across study groups during the study.

**Detection bias**

Detection bias relates to how the exposure and outcome are assessed.

1. Can we be confident in the exposure characterisation? (All)

Definitely low risk of bias (++)

There is a direct evidence that the exposure was independently characterized. Independent verification of the exposure is possible if dosimeter was used or the machine was properly calibrated.

The authors should also mention the ionization source, the dose-rates and time for ionization.

Probably low risk of bias (+)
There is no reporting of use of dosimeters or no calibrations were performed beforehand but the authors mention the ionization source and the dose-rates.

Probably high risk of bias (-)
Only the dose received is mentioned along with the ionization source.

There is neither a mention of dosimeters nor of calibrations.

Definitely high risk of bias (--)
The authors mention neither of the following: ionization source, dosimeters, calibration, dose-rate and the dose received is provided in a range between two doses (e.g. 4-5 Gy)

1. Can we be confident in the outcome assessment? (all)

Definitely low risk of bias (++)
There is a direct evidence of use of valid and reliable methods to access the outcomes. The outcomes were measured with the same method and time after exposure along all groups and has been validated by another valid method (eg. mass-spectrometry proteomic data has been validated with western blots).

There is a direct evidence that the outcome accessor was adequately blinded to study groups.

Probably low risk of bias (+)
There is an indirect evidence of use of valid and reliable methods to access the outcomes and the accessor was adequately blinded to the study groups.

Probably high risk of bias (-)
There is an indirect evidence of use of unreliable methods to assess the outcome, including a lack of validation of the reported outcomes and self-reporting.

There is an indirect evidence of the outcome accessor being not adequately blinded to study groups.

There is an indirect evidence of varying lengths of time between exposure and outcome across study groups.

Definitely high risk of bias (--)
There is a direct evidence of use of unreliable methods, including self-reporting to access the outcome and there is a lack of validation for the reported outcomes.

There is a direct evidence that the outcome accessor not being adequately blinded to study groups.

There is a direct evidence of varying lengths of time between exposure and outcome across study groups.

**Selective reporting bias**

Selective reporting refers to the presence of bias when pre-specified outcomes are not reported.

1. Were all measuring outcomes reported? (All)

Definitely low risk of bias (++)

There is a direct evidence that the outcomes of the study as outlined in protocol, methods and/or introduction have been reported.

Probably low risk of bias (+)
There is an indirect evidence that all outcomes of the study, as outlined, have been reported.

Probably high risk of bias (-)
There is an indirect evidence that all outcomes of the study have not been reported.

Definitely high risk of bias (--)
There is a direct evidence that all outcomes of the study have not been reported.

**Other biases**

1. Were there any other potential threats to internal validity? (e.g. Statistical methods) (All)

   Definitely low risk of bias (++)
   There is a direct evidence of adherence to study protocol and the authors do a proper statistical adjustments for comparing outcomes.

The authors mention that there is no conflict of interest.

Probably low risk of bias (+)
There is an indirect evidence of adherence to study protocol and the authors mention statistical adjustments for comparing outcomes (e.g. the authors mention that a corrected p-Value is provided but fail to mention what kind of correction was performed.

There is an indirect inference to absence of any conflict of interests.

Probably high risk of bias (-)
There is an indirect evidence of non-adherence to study protocol and the authors do mention statistical adjustments for comparing outcomes.

It can be inferred that there exists an conflict of interests (eg. study for a drug that reduces side effects during RT is funded by the drug company)

Definitely high risk of bias (--)
There is a direct evidence of non-adherence to study protocol.

The authors mention a conflict of interest.

There is a mention of unintended co-exposures.
