# Supplementary material for: Ionizing Radiation Protein Biomarkers in Normal Tissue and Their Correlation to Radiosensitivity: A Systematic Review
Source: J Pers Med. 2021 Feb 19;11(2):140. doi: 10.3390/jpm11020140 (PMC7922485; doi:10.3390/jpm11020140)
Supplement: Supplementary file 1 [file jpm-11-00140-s001.zip › SWiM checklist.docx]

SWiM checklist as reported byCampbell et al., Synthesis without meta-analysis(SWiM) in systematic reviews: reporting guideline BMJ 2020; 368 :16890

| SWiM reporting item | Item Description | Page in manuscript where item is reported |
| --- | --- | --- |
| Methods | | |
| 1 Grouping studies for synthesis | 1a) Provide a description of, and rationale for, the groups used in the synthesis (eg, groupings of populations, interventions, outcomes, study design) | Page 4, Lines 142-145 |
|  | 1b) Detail and provide rationale for any changes made subsequent to the protocol in the groups used in the synthesis | N/A |
| 2. Describe the standardised metric and transformation methods used | Describe the standardised metric for each outcome. Explain why the metric(s) was chosen and describe any methods used to transform the intervention effects, as reported in the study, to the  standardised metric, citing any methodological guidance consulted | Page 4, Lines 147-150 |
| 3. Describe the synthesis methods | Describe and justify the methods used to synthesise the effects for each outcome when it was not possible to undertake a meta-analysis of effect estimates | Page 4, Lines 152-153 |
| 4. Criteria used to prioritise results for summary and synthesis | Where applicable, provide the criteria used, with supporting justification, to select the particular studies, or a particular study, for the main synthesis or to draw conclusions from the synthesis (eg, based on study design, risk of bias assessments, directness in relation to the review question) | Page 5, Lines 199-201 |
| 5. Investigation of heterogeneity in reported effects | State the method(s) used to examine heterogeneity in reported effects when it was not possible to undertake a meta-analysis of effect estimates and its extensions to investigate heterogeneity | Page 4, Lines 139-140 |
| 6. Certainty of evidence | Describe the methods used to assess the certainty of the synthesis findings | Page 4, Lines 155-162 |
| 7. Data presentation methods | Describe the graphical and tabular methods used to present the effects (eg, tables, forest plots, harvest plots) | Page 4, Lines 139-140 |
|  | Specify key study characteristics (eg, study design, risk of bias) used to order the studies, in the text and any tables or graphs, clearly referencing the studies included | Page 5, Lines 199-201 |
| **Results** |  |  |
| Reporting results | For each comparison and outcome, provide a description of the synthesised findings and the certainty of the findings. Describe the result in language that is consistent with the question the synthesis addresses, and indicate which studies contribute to the synthesis | Page 6, Lines 216-217; Lines 243-245, Tables 2-6; Page 5-9 |
| **Discussion** | | |
| Limitations of the synthesis | Report the limitations of the synthesis methods used and/or the groupings used in the synthesis and how these affect the conclusions that can be drawn in relation to the original review question | Page 9-10, Lines 383-415 |
